# Impaired Self-Monitoring of Inner Speech in Schizophrenia Patients with Verbal Hallucinations and in Non-clinical Individuals Prone to Hallucinations

**DOI:** 10.3389/fpsyg.2016.01381

**Published:** 2016-09-14

**Authors:** Gildas Brébion, Christian Stephan-Otto, Susana Ochoa, Mercedes Roca, Lourdes Nieto, Judith Usall

**Affiliations:** Research and Development Unit, Parc Sanitari Sant Joan de Déu and Centro de Investigación Biomédica en Red de Salud MentalBarcelona, Spain

**Keywords:** verbal hallucinations, verbal memory, self-monitoring, schizophrenia, psychosis continuum

## Abstract

**Background:** Previous research has shown that various memory errors reflecting failure in the self-monitoring of speech were associated with auditory/verbal hallucinations in schizophrenia patients and with proneness to hallucinations in non-clinical individuals.

**Method:** We administered to 57 schizophrenia patients and 60 healthy participants a verbal memory task involving free recall and recognition of lists of words with different structures (high-frequency, low-frequency, and semantically organisable words). Extra-list intrusions in free recall were tallied, and the response bias reflecting tendency to make false recognitions of non-presented words was computed for each list.

**Results:** In the male patient subsample, extra-list intrusions were positively associated with verbal hallucinations and inversely associated with negative symptoms. In the healthy participants the extra-list intrusions were positively associated with proneness to hallucinations. A liberal response bias in the recognition of the high-frequency words was associated with verbal hallucinations in male patients and with proneness to hallucinations in healthy men. Meanwhile, a conservative response bias for these high-frequency words was associated with negative symptoms in male patients and with social anhedonia in healthy men.

**Conclusion:** Misattribution of inner speech to an external source, reflected by false recollection of familiar material, seems to underlie both clinical and non-clinical hallucinations. Further, both clinical and non-clinical negative symptoms may exert on verbal memory errors an effect opposite to that of hallucinations.

## Introduction

Inner speech, also referred to as verbal thinking, might have a central role in the self-regulation of cognition, behavior, and social interactions. Its origin and functional significance have been theorized by the developmental psychologist Vygotsky. “Via a mechanism of internalization, linguistically mediated social exchanges (such as those between the child and a caregiver) are transformed, in Vygotsky’s model, into an internalized “conversation” with the self” (Alderson-Day and Fernyhough, 2015, p. 932). Inner speech would therefore be dialogic and social in nature, and its disruption may lead to communication and psychiatric disorders.

Verbal hallucinations, which are a common manifestation of schizophrenia, have been proposed to be associated with failure in the self-monitoring of inner speech. Notably, several studies have reported that schizophrenia patients with auditory/verbal hallucinations tend to misattribute their own verbal production to an external source (Bentall et al., 1991; Johns et al., 2001, 2006; Woodward et al., 2007; Costafreda et al., 2008), a bias also observed in patients with delusions (Johns et al., 2006; Anselmetti et al., 2007; Costafreda et al., 2008) and in non-clinical individuals prone to hallucinations (Larøi et al., 2004) or to delusions (Allen et al., 2006). In addition, patients with auditory hallucinations demonstrated an increased tendency to remember silently read words as having been spoken aloud (Franck et al., 2000) and imagined words as having been heard (Brunelin et al., 2006), a kind of error also reported in non-clinical individuals prone to hallucinations (Rankin and O’Carroll, 1995). Confusion between said and thought information was also observed in schizophrenia patients with thought disorganization (Harvey, 1985; Nienow and Docherty, 2004).

Failure in the self-monitoring of inner speech may also be reflected by certain types of commission errors in verbal memory tasks. One type of commission error consists in making extra-list intrusions in the free recall of lists of words, i.e., recalling words that were not included in the target list. In a previous study, we observed that extra-list intrusions were associated with verbal hallucinations in schizophrenia patients (Brébion et al., 2009) and with proneness to hallucinations in non-clinical individuals (Brébion et al., 2010). Extra-list intrusions have also been found to be associated with delusions (Brébion et al., 1999; Rocca et al., 2006; Stip et al., 2007; Bhatt et al., 2010) and thought disorganization (Brébion et al., 1999; Subotnik et al., 2006; Fridberg et al., 2010) in schizophrenia samples, as well as with delusional ideation in healthy samples (Laws and Bhatt, 2005; Dehon et al., 2008; Bhatt et al., 2010). However, they were unrelated to a positive symptomatology score in the data of Heinrichs and McDermid Vaz (2004), and they were unrelated to either hallucinations or delusions in those of Fridberg et al. (2010).

Another type of commission error is the false recognition of non-target words in recognition tasks, in which participants are required to identify the previously presented words among equivalent distractors. We observed in two distinct schizophrenia samples that a global hallucination score was associated with liberal response bias, i.e., with a tendency to make such false recognitions (Brébion et al., 1998, 2005). However, no specific association with verbal hallucinations has yet been established in patients. With regard to healthy individuals, participants scoring high vs. low on an auditory hallucination proneness scale did not demonstrate any response bias difference in the study of McKague et al. (2012). Nevertheless, our group evinced an association between hallucination proneness score and liberal response bias for both a non-organisable and a semantically organisable list of words (Brébion et al., 2010). Using a different paradigm, Sugimori et al. (2011) and Kanemoto et al. (2013) demonstrated that proneness to auditory hallucinations was associated with false recognitions of lures. False recognitions might not be specific to people presenting with hallucinations, though. In the study of Ragland et al. (2003), delusions and thought disorders, rather than hallucinations, were indeed associated with a liberal response bias in word recognition. Moritz et al. (2003, 2005) observed in two separate studies an association between thought disorders and false recognitions of new words that were related to the target words. Lastly, proneness to delusions in a non-clinical sample was found to be associated with increased rates of false recognitions of new words, and confidence in their alleged presentation (Laws and Bhatt, 2005).

A less expected and more challenging finding in our data was that these commission errors in verbal memory were inversely associated with negative symptoms in patients. Higher ratings of affective flattening and anhedonia were associated with fewer extra-list intrusions (Brébion et al., 1999, 2009). An inverse association between number of intrusions and negative symptoms was similarly reported by Heinrichs and McDermid Vaz (2004), although not by Rocca et al. (2006) or Fridberg et al. (2010). Turetsky et al. (2002) observed that a subgroup of patients with higher ratings of affective flattening than the other subgroup presented fewer intrusions. In our previous work, affective flattening and anhedonia were also associated with a more conservative response bias in a word recognition task, i.e., with a decreased tendency to make false recognitions of non-target words (Brébion et al., 2005). Paz-Alonso et al. (2013) similarly reported an inverse association between negative symptom scores and rates of false recognitions of words, although no association with any negative symptom was evinced by Ragland et al. (2003). Inverse associations with commission errors in certain studies could mean that negative symptoms are associated with intensification of inhibitory mechanisms, as suggested by Heinrichs and McDermid Vaz (2004). However, such associations do not appear to be restricted to the memory errors that might result from poor inhibition. Errors stemming from self-monitoring failure appear to be involved as well. Indeed, we observed that patients with blunted affect made fewer misattributions of their own verbal production to an external source than did other patients (Brébion et al., 2002). Stirling et al. (2001) used a self-monitoring task involving the recognition of one’s own drawings among others and reported that, while positive symptoms were associated with more errors, negative symptoms were associated with fewer. Very recently, a self-monitoring study conducted in an acute psychiatric sample revealed that positive symptoms were associated with tendency to misattribute self-produced verbal items to the experimenter, while negative symptoms were associated with tendency to misattribute to oneself the items produced by the experimenter (Chiu et al., 2016). In non-clinical populations no association between negative schizotypy and decreased rates of memory errors has yet been reported, as far as we know. However, Minor et al. (2011) observed that negative schizotypy was associated with less atypicality in the production of category exemplars.

The first objective of this study was to further explore the association between verbal hallucinations and impaired self-monitoring of inner speech in schizophrenia. We used a verbal memory task that yielded extra-list intrusions and false recognitions. Considering that errors in the self-monitoring of speech have also been reported in patients presenting delusions and/or thought disorganization, associations with these symptoms were investigated as well so as to better identify what is specific to verbal hallucinations. To refine previous findings of an association between hallucinations and false recognitions of non-target words, we manipulated the structure of the lists of words to be learned. The usage frequency of the target words might be a relevant parameter as far as verbal hallucinations are concerned. Indeed, if the false recognitions stem from confusion between inner speech and target words, one might assume that they apply mostly to familiar material, reflected by high-frequency words. Only lists of common words were used in Brébion et al. (1998), while in Brébion et al. (2005) we used lists of mixed high- and low-frequency words. In the current study we contrasted pure lists of high-frequency words and pure lists of low-frequency words. Another potentially relevant factor is the semantic organization of the list, since semantic abnormalities have been found to be associated with delusions (Rossell et al., 2010; Cameron et al., 2014) and thought disorganization (Goldberg et al., 1998; Kerns and Berenbaum, 2002). We therefore contrasted semantically organisable and non-organisable lists of words. Our hypothesis was that verbal hallucinations were specifically associated with false recognitions of common words but that this association was not affected by semantic organization. On the other hand, delusions and thought disorganization were both expected to show different associations with the response bias as a function of semantic organization. With regard to extra-list intrusions, they were expected, on the basis of previous findings, to be associated with verbal hallucinations, delusions, and thought disorganization. However, entering these three clinical symptoms altogether in a regression analysis would enable us to ascertain whether the association with verbal hallucinations is genuine. In agreement with our previous findings and a few others, we also expected negative symptoms to be associated with decreased rates of intrusions and false recognitions in patients.

All the above-mentioned errors in the self-monitoring of speech, which were found to be associated with hallucinations in patients, have also been found to be associated with proneness to hallucinations in the general population. This argues in favor of a continuum between clinical hallucinations and non-pathological psychological experiences (van Os et al., 2009; Johns et al., 2014). The second objective of our study was to strengthen the theory of this continuum by demonstrating that the mechanisms uncovered by our verbal memory task underlie both non-clinical and clinical hallucinations. We administered the task to healthy individuals and assessed their propensity to non-clinical hallucinations and delusions. Negative schizotypy was assessed as well by means of a social anhedonia scale. In agreement with our previous study of healthy participants and two others (Brébion et al., 2010; Sugimori et al., 2011; Kanemoto et al., 2013), we expected proneness to hallucinations to be associated with increased rates of extra-list intrusions and false recognitions of non-target words. The manipulation of word frequency in the lists of words was hypothesized to produce similar effects in the healthy sample as it would in patients. Associations with delusion proneness were expected as well on the basis of previous findings (Laws and Bhatt, 2005; Dehon et al., 2008; Bhatt et al., 2010). Whether social anhedonia is inversely associated with the rates of false recollections, similarly to negative symptoms, was investigated.

Our previous work and other recent studies have revealed differences between male and female patients in the way clinical factors relate to performance in memory and various other cognitive domains (Karilampi et al., 2011; Han et al., 2012; Brébion et al., 2013b, 2015; Mendrek and Mancini-Marïe, 2016). This may point to the necessity to develop different rehabilitation strategies in men than in women. We therefore conducted the analyses separately in men and in women to determine whether the expected associations of the verbal memory errors with the target clinical and non-clinical symptoms were similarly observed in each sex group.

## Materials and Methods

### Participants

Fifty-seven inpatients with schizophrenia (DSM-IV criteria) were recruited from the Parc Sanitari Sant Joan de Déu network of mental health services in Barcelona, Spain (36 males, 21 females). The diagnosis was made by consensus on the basis of DSM IV criteria by two experienced psychiatrists who used patient histories and chart reviews. The patients suffered from chronic schizophrenia, with disease duration of over 2 years. The inclusion criteria were age between 18 and 70 years, fluency in Spanish, and the ability to provide informed consent. The exclusion criteria were current or recent alcohol or drug abuse (DSM-IV criteria), organic mental disorders, intellectual disability, history of brain injury, dementia, and current severe physical disease. All the patients were receiving antipsychotic medication and were at a stabilized dose at the time of testing. Male and female patients were not significantly different with respect to age, education level or verbal IQ. However, females presented significantly less illness duration than did males (**Table 1**).

**Table 1:** Socio-demographic information and rating scale scores for schizophrenia patients and healthy controls (means and standard deviations).

	Schizophrenia patients		Healthy controls	
	36 men	21 women	35 men	25 women
Age	47.4 (10.3)	44.7 (11.0)	47.1 (8.4)	43.8 (10.1)
Education level^1^	3.1	3.8	4.6	4.9
Verbal IQ^2^	91	91	98	95
Illness duration	21.4 (12.0)	14.0 (11.7)		
Hallucinations	6.4 (5.0)	8.9 (6.2)		
Verbal hallucinations	2.4 (2.2)	3.4 (3.4)		
Delusions	12.6 (8.3)	12.3 (7.8)		
Disorganization	8.9 (5.7)	7.8 (7.1)		
Negative symptoms	26.8 (12.5)	17.5 (12.4)		
LSHS			6.1 (4.6)	4.6 (2.9)
PDI			7.6 (5.7)	5.4 (4.2)
Social anhedonia			10.7 (5.1)	11.0 (5.5)

^1^*The scale used was: 1 = no studies; 2 = uncompleted primary studies; 3 = completed primary studies; 4 = high school uncompleted; 5 = high school completed; 6 = uncompleted undergraduate studies; 7 = bachelor’s or master’s degree; 8 = doctorate*.

^2^*Corresponding estimated IQs for the *Test de Acentuación de Palabras* scores (Gomar et al., 2011)*.

Sixty healthy control participants were recruited from the community (35 males, 25 females, **Table 1**). They were screened by telephone interview to rule out current or recent alcohol abuse, drug abuse, and psychiatric disease, as well as severe current non-psychiatric disease. Patients and controls were not significantly different in age or sex distribution. However, the level of education was significantly higher in the control group, as was the score on the vocabulary test (Test de Acentuación de Palabras), a Spanish equivalent of the National Adult Reading Test (NART), used to assess verbal IQ. Ethical approval for the study was obtained from the Research and Ethics Committee of our institution. Subjects provided written informed consent after receiving a full explanation of the study.

### Clinical Ratings

Positive and negative symptoms were assessed in patients by means of the Spanish version of the SAPS and SANS (Peralta and Cuesta, 1999). Clinical assessment was conducted shortly after the completion of the task by a trained clinical psychologist who was blind to the hypotheses of the study. Scores for hallucinations, delusions, and thought disorganization were tallied. In addition, a verbal hallucination score was computed by adding up the scores obtained on the 2nd and 3rd items of the hallucination subscale (‘voices commenting’ and ‘voices conversing’). A negative symptom score was computed by adding up the scores obtained for the following symptoms: affective flattening, alogia, avolition/apathy, and anhedonia. The negative symptom score was significantly lower in female than in male patients (**Table 1**).

The healthy control participants were administered Spanish adaptations of self-questionnaires assessing proneness to hallucinations (Launay-Slade Hallucination Scale – LSHS; Launay and Slade, 1981), proneness to delusions (Peters Delusion Inventory – PDI; Peters et al., 2004), and social anhedonia (revised social anhedonia scale; Fonseca-Pedrero et al., 2009). Male and female controls were not significantly different in any of these measures.

### Material

Six lists of 16 concrete words, all equivalent with respect to the total number of syllables, were constructed and printed out. Two of the lists were made up of high-frequency words, with an occurrence ≥44 per million (average word frequency: 189.5, for one list and 189.6 for the other). Two other lists were made up of low-frequency words, with an occurrence ≤16 per million (average word frequency: 9.1 for one list and 9.4 for the other; Algarabel et al., 1988). The last two lists were made up of words randomly distributed but organisable into four semantic categories: *types of vehicle*, *animals*, *fruits*, and *clothes* (average word frequency: 66.8 for one list and 64.3 for the other). Three recognition sheets were prepared including, in random order, the 32 high-frequency words for one sheet, the 32 low-frequency words for another one, and the 32 semantically organisable words for the last one. Within each pair of equivalent lists, one was used as target and the other as distractor. The use of each list as target or distractor was counterbalanced among subjects, as was the order of presentation of the three target lists.

### Procedure

One high-frequency, one low-frequency, and one semantically organisable list were administered, all with identical procedure. The printed list was presented for 45 s and the participants were instructed to study it in order to memorize it. After a delay of a few minutes filled with unrelated tasks, they were provided with a blank sheet and required to write down all the words they could remember, in any order and without any time limit. Following the recall task they were presented with the corresponding recognition sheet and required to circle the words they could recognize from the previously learned list. Then the following target list was presented.

The number of correctly recalled words and extra-list intrusions in free recall was tallied for each list. In the recognition task, the number of correctly recognized target words and false recognitions of non-target words were tallied. These two measures were used to compute, for each list, the recognition index Pr, reflecting the ability to discriminate the target words from the distractors, and the response bias index Br, reflecting the tendency to make false recognitions of non-presented words in case of uncertainty (Corwin, 1994). Global scores for number of recalled words, number of intrusions, Pr recognition index, and response bias Br were averaged across the three lists. The numbers of intrusions did not follow normal distribution in either group and they were subjected to square-root transformation before data analysis, as were the hallucination and delusion proneness scores for the healthy sample. Data for a few Br values were discarded because of maximal recognition score (**Table 2**).

**Table 2:** Scores observed in the free recall and recognition tasks in schizophrenia patients and healthy controls as a function of the type of list (means and standard deviations).

		Schizophrenia patients		Healthy controls	
		36 men	21 women	35 men	25 women
**Number of words recalled**	High-frequency list	1.8 (1.8)	2.6 (2.1)	4.3 (2.4)	4.5 (2.2)
	Low-frequency list	1.7 (1.6)	2.0 (1.7)	3.2 (1.8)	3.8 (2.3)
	Organisable list	2.1 (2.2)	2.7 (2.1)	4.8 (2.0)	5.7 (2.1)
**Number of extra-list intrusions**	High-frequency list	1.2 (1.9)	0.57 (0.87)	0.60 (1.0)	0.60 (0.82)
	Low-frequency list	0.78 (1.3)	0.29 (0.46)	0.60 (0.81)	0.24 (0.44)
	Organisable list	0.75 (1.5)	0.43 (0.81)	0.43 (0.78)	0.24 (0.52)
**Recognition index Pr**	High-frequency list	0.40 (0.21)	0.37 (0.24)	0.51 (0.22)	0.55 (0.17)
	Low-frequency list	0.45 (0.24)	0.48 (0.23)	0.60 (0.16)	0.67 (0.15)
	Organisable list	0.38 (0.18)	0.36 (0.23)	0.59 (0.19)	0.60 (0.16)
**Response bias Br^1^**	High-frequency list	0.25 (0.22)	0.20 (0.18)	0.27 (0.19)	0.33 (0.24)
	Low-frequency list	0.20 (0.18)	0.19 (0.15)	0.34 (0.18)	0.34 (0.21)
	Organisable list	0.34 (0.25)	0.22 (0.20)	0.34 (0.23)	0.44 (0.18)

^1^*High-frequency list: Br value is missing for one healthy participant; low-frequency list: Br value is missing for two patients and for one healthy participant; semantically organisable list: Br value is missing for two healthy participants*.

## Results

### Verbal Memory Scores

The verbal memory scores observed in schizophrenia patients and healthy controls, separately in men and women, are presented in **Table 2**. ANOVAs were conducted on the numbers of words recalled, numbers of extra-list intrusions, recognition indices Pr, and response bias Br, with the type of list (high-frequency, low-frequency, semantically organisable) as a within-subject factor, and group (patients, healthy controls) and sex as between-subject factors.

#### Number of Words Recalled

A highly significant effect of group was revealed, reflecting the impaired recall performance in patients [*F*(1,113) = 61.9, *p* < 0.0001]. It should be noted that this effect remained highly significant when education level and verbal IQ were covariated to adjust for group differences in these measures [*F*(1,111) = 35.0, *p* < 0.0001]. There was also a marginally significant effect of sex [*F*(1,113) = 3.9, *p* = 0.051], indicating that women recalled more words than men, without any interaction with group (*F* near zero). The effect of the type of list was significant [*F*(2,226) = 13.9, *p* < 0.0001], with a significant group × type of list interaction [*F*(2,226) = 3.9, *p* < 0.025].

#### Number of Extra-List Intrusions

The effect of group was not significant [*F*(1,113) = 1.1, ns]. A trend for an effect of sex emerged [*F*(1,113) = 3.4, *p* < 0.07], revealing that men tended to experience more intrusions than women, without any interaction with group (*F* < 1). It should be noted that this trend was still observed when the total number of words recalled was controlled [*F*(1,112) = 2.9, *p* < 0.10). The effect of the type of list was significant [*F*(2,226) = 3.8, *p* < 0.025], without any interaction with group (*F* < 1). Examination of the means revealed that, in both groups, more intrusions were experienced in the recall of the high-frequency list.

#### Pr Recognition Index

A highly significant effect of group indicated that patients were impaired in word recognition [*F*(1,113) = 35,8, *p* < 0.0001]. Again, this effect remained highly significant when education level and verbal IQ were controlled [*F*(1,111) = 22.0, *p* < 0.0001]. No effect of sex (*F* < 1) or interaction between group and sex (*F* < 1) was observed. The effect of the type of list was highly significant [*F*(2,226) = 11.1, *p* < 0.0001] without any significant interaction with group [*F*(2,226) = 1.9, ns]. Examination of the scores revealed that the low-frequency list was better recognized in each group than were the other two lists.

#### Response Bias Br

A significant effect of group was observed [*F*(1,107) = 11.6, *p* < 0.001], indicating that the patients were more conservative than the healthy controls. The main effect of sex was not significant (*F* near zero), but there was a significant effect for the type of list [*F*(2,214) = 11.1, *p* < 0.0001] as well as a significant three-way group × sex × type of list interaction [*F*(2,214) = 4.1, *p* < 0.025]. Follow-up *t*-test analyses revealed that female patients were significantly more conservative than healthy women for each of the three lists. Male patients, in contrast, were equivalent to healthy men for the high-frequency and semantically organisable lists (*t* < 1 in both cases), while they were significantly more conservative than healthy men for the low-frequency list [*t*(67) = 3.25, *p* < 0.002].

An exploratory investigation of the potential effect of illness duration –a clinical factor that differentiated male and female patients– on memory performance revealed that illness duration was strongly associated with the number of recalled words (*r* = -0.51, *p* < 0.0001), but was unrelated to the Pr recognition index (*r* near zero).

### Associations between Extra-List Intrusions and Rating Scales

Regression analyses were conducted on the total number of extra-list intrusions.

#### Schizophrenia Patients

In the patient group the independent variables were the total number of words recalled, and the verbal hallucination, delusion, thought disorganization, and negative symptom scores. Illness duration was entered as well because of its strong association with free recall. In the 21 women, the thought disorganization score made a near zero contribution to the number of intrusions and it was removed from the predictors. The regression analysis recomputed with the remaining variables showed that illness duration was significantly and positively associated with the number of intrusions (β = 0.74, *p* < 0.05), while a trend for a negative association with the delusion score emerged (β = -0.64, *p* = 0.10). In the 36 men, the number of words recalled, delusion score, and illness duration did not make any contribution to the number of intrusions and they were removed from the model. The regression analysis recomputed in the male subsample with the remaining three predictors yielded a significant model [*F*(3,35) = 4.5, *p* < 0.01; *R*^2^ = 0.30]. Both verbal hallucinations (β = 0.48, *p* < 0.01) and thought disorganization (β = 0.33, *p* < 0.05) were positively associated with the number of intrusions, while negative symptoms were inversely associated with it (β = -0.41, *p* < 0.05). It should be noted that, in male patients, the delusion score was significantly and positively associated with the number of intrusions in a simple correlational analysis (*r* = 0.43, *p* < 0.01).

#### Healthy Participants

In the healthy control group the regression analysis included the total number of words recalled and the scores on the Launay-Slade Hallucinations, Peters Delusion Inventory, and social anhedonia scales. No association was observed in the 35 men (β near zero for the three rating scales) or the 25 women (*p* > 0.40 for all variables). Following procedure used with two previous samples (Brébion et al., 2009, 2010), we recomputed the regression analysis in the subsample of 37 healthy male and female participants who made at least one extra-list intrusion, after adding sex to the predictors. In the same way as in the male patients, the recall score and delusion proneness score were unrelated to the number of intrusions and they were removed from the predictors. The regression analysis including hallucination proneness score, social anhedonia score, and sex as predictors [*F*(3,36) = 3.2, *p* < 0.05; *R*^2^ = 0.23] revealed that the total number of intrusions was positively associated with hallucination proneness (β = 0.37, *p* < 0.05). The inverse association with social anhedonia did not reach statistical significance (β = -0.24, *p* < 0.15).

### Associations between Response Bias and Rating Scales

Similar regression analyses were conducted on the global response bias in each group.

#### Schizophrenia Patients

In patients, the independent variables were the global Pr index and the verbal hallucination, delusion, thought disorganization, and negative symptom scores. In the subgroup of 21 female patients, thought disorganization and negative symptom scores made a near zero contribution to the response bias and they were removed from the predictors. When verbal hallucination score, delusion score, and Pr index were entered in the regression analysis, delusion score made a significant negative contribution to the response bias, indicating that higher rates of delusions were associated with a decreased tendency to make false recognitions of non-target words. An analysis of each list indicated that the inverse association with delusions was significant for the high-frequency and the low-frequency lists, but not for the semantically organisable list (β = -0.28, *p* = 0.30). These results are reported in **Table 3**.

**Table 3:** Regression analyses in the 21 female schizophrenia patients.

	Verbal hallucinations	Delusions	Recognition index Pr	*R*^2^	*F* test	*p*-value
**Global Br**	0.25	**-0.63^∗∗^**	0.24	0.34	2.97	0.07
**Br high-frequency**	0.34	**-0.60^∗^**	0.06	0.23	1.65	ns
**Br low-frequency**	0.40	**-0.59^∗^**	0.15	0.24	1.82	ns
**Br organisable**	-0.10	-0.28	0.32	0.26	1.99	ns

*Associations of verbal hallucinations and delusions with the response bias in each type of list (β coefficient). ^∗^*p* < 0.05; ^∗∗^*p* < 0.025*.

*Bold values indicate associations significant, or tending to be significant, with the clinical symptoms of interest*.

In the 36 male patients, the delusion score made a near zero contribution to the response bias, and it was removed from the predictors. The regression analysis conducted with the four remaining predictors (**Table 4**) showed that thought disorganization made a significant, and verbal hallucinations a marginally significant (*p* < 0.06), positive contribution to the response bias. Analysis of each list revealed that verbal hallucinations were associated with increased rates of false recognitions in the high-frequency and the semantically organisable lists but not in the low-frequency list, as predicted. Thought disorganization was associated with increased rates of false recognitions in the high-frequency list, and, at a trend level, in the low-frequency list. The expected inverse association with negative symptoms was only observed at a trend level in the high-frequency list (*p* < 0.06). It should be noted that if correlations were conducted with the delusion score after controlling only for Pr, a positive association between delusion score and response bias in the high-frequency list was observed in male patients (β = 0.36, *p* < 0.05).

**Table 4:** Regression analyses in the 36 male schizophrenia patients.

	Verbal hallucinations	Thought disorganization	Negative symptoms	Recognition index Pr	*R*^2^	*F* test	*p*-value
**Global Br**	**0.31^+^**	**0.39^∗^**	-0.16	0.58^∗∗∗^	0.35	4.21	0.01
**Br high-frequency**	**0.36^∗∗^**	**0.48^∗∗∗^**	**-0.32^+^**	0.61^∗∗∗^	0.44	6.16	0.001
**Br low-frequency**	-0.11	**0.34^+^**	0.08	0.55^∗∗∗^	0.26	2.54	0.07
**Br organisable**	**0.39^∗^**	0.22	-0.10	0.35^+^	0.22	2.20	0.10

*Associations of verbal hallucinations, thought disorganization, and negative symptoms with the response bias in each type of list (β coefficient). ^+^*p* < 0.10; ^∗^*p* < 0.05; ^∗∗^*p* < 0.025; ^∗∗∗^*p* < 0.005*.

*Bold values indicate associations significant, or tending to be significant, with the clinical symptoms of interest*.

#### Healthy Participants

In the healthy control group the global Pr index and the scores on the Launay-Slade Hallucinations, Peters Delusion Inventory, and social anhedonia scales were the independent variables. In the 25 women, no association with hallucination proneness (β = -0.09), delusion proneness (β = 0.17), or social anhedonia (β = -0.04) was observed. In the 35 men, a significant positive association with hallucination proneness emerged. As was the case in the male patient group, the delusion proneness score made a near zero contribution to the response bias, and it was removed from the predictors. The regression analysis was recomputed with the other three variables (**Table 5**). Analysis of each list revealed that proneness to hallucinations was significantly associated with liberal response bias in the high-frequency and the semantically organisable lists but not the low-frequency list, similarly to what was observed in male patients. Social anhedonia was associated with a more conservative response bias in the high-frequency list and, at a trend level, in the semantically organisable list.

**Table 5:** Regression analyses in the 35 healthy male participants.

	Hallucination proneness	Social anhedonia	Recognition index Pr	*R*^2^	*F* test	*p*-value
**Global Br**	**0.43^∗∗∗^**	-0.27	0.32^+^	0.29	4.27	0.015
**Br high-frequency**	**0.44^∗∗∗^**	**-0.42^∗∗^**	0.20	0.32	4.76	0.01
**Br low-frequency**	0.23	0.10	0.45^∗∗^	0.19	2.38	0.09
**Br organisable**	**0.40^∗^**	**-0.33^+^**	0.18	0.24	3.09	0.05

*Associations of hallucination proneness and social anhedonia with the response bias in each type of list (β coefficient). ^+^*p* < 0.10; ^∗^*p* < 0.05; ^∗∗^*p* < 0.025; ^∗∗∗^*p* < 0.015*.

*Bold values indicate associations significant, or tending to be significant, with the clinical symptoms of interest*.

## Discussion

### Verbal Hallucinations

The main objective of this study was to better characterize the relation between verbal hallucinations and impaired self-monitoring of inner speech in patients with schizophrenia. We focused on two types of commission error in verbal memory tasks, namely the extra-list intrusions in free recall and the false recognitions of non-presented words in recognition, both assumed to stem from self-monitoring failure.

As expected, our data revealed that both of these errors were associated with verbal hallucinations, although this was observed only in the male patients. Verbal hallucinations were associated with an increased number of extra-list intrusions in the recall of lists of words, corroborating our previous study (Brébion et al., 2009). This observation suggests that inner speech was not recognized as self-generated –a cognitive failure associated with auditory hallucinations (Waters et al., 2012b)– and that it was mixed with the content of the recall list. Verbal hallucinations were also associated with false recognitions of non-presented words and, as expected, this association pertains only to the common words. Again, this finding is compatible with the theory of inner speech being interpreted as an external perception (Allen et al., 2007; Jones, 2010; Moseley et al., 2013, 2016; Alderson-Day and Fernyhough, 2015). On the other hand, the semantic structure of the list of words did not seem to have any impact on this association.

The fact that associations with intrusions and response bias in our data emerged only in male patients suggests that women are less vulnerable than men to this type of cognitive impairment. Barkus et al. (2011) observed a more liberal response bias in healthy men than in healthy women in an auditory signal detection task. In our data, men from both groups tended to experience more intrusions in free recall than did their female counterparts. A recent study revealed that, in schizotypal women, high levels of estradiol were associated with decreased rates of false recognitions of non-presented words (Hodgetts et al., 2015). Although verbal hallucinations are also observed in women, they might occur through mechanisms others than disruption in the self-monitoring of inner speech. Other types of source-monitoring deficit may be involved. Indeed, previous research from our group indicated that auditory hallucinations were associated with failure to process or remember the spatiotemporal context of the events (Brébion et al., 2012). Another line of research emphasizes the role of metacognitive disturbances in the formation of this symptom (Salvatore et al., 2016). Notably, verbal hallucinations may arise from dysfunction in certain processes that enable reflection about oneself. This includes faulty sense of ownership and agency, which may lead to the conclusion that internal experiences such as thoughts have an external origin (Dimaggio et al., 2009). Impairment in the capacity to form complex and integrated representations of self and others might also contribute to difficulty in recognizing one’s own thoughts and intentions (Lysaker and Dimaggio, 2014). Abnormalities in emotional arousal and emotional awareness also need to be taken into account since emotion plays a prominent role in the formation and maintenance of verbal hallucinations (Serper and Berenbaum, 2008; Badcock et al., 2011; Waters et al., 2012a). Women might be relatively protected against dysfunction in the self-monitoring of inner speech, but might be, on the other hand, particularly vulnerable to certain types of metacognitive or source-monitoring deficits. Potential gender-related differences in the cognitive underpinnings of verbal hallucinations should be further investigated as they may have implications for the cognitive remediation of this symptom.

### Delusions

In male patients, delusions were significantly associated with an increased number of extra-list intrusions in a correlational analysis, which corroborates several studies (Brébion et al., 1999; Laws and Bhatt, 2005; Rocca et al., 2006; Stip et al., 2007; Dehon et al., 2008; Bhatt et al., 2010). When only recognition accuracy was controlled, delusions were also associated with liberal response bias, in agreement with Ragland et al. (2003) and Laws and Bhatt (2005). However, both associations disappeared when regression analyses involving the other clinical symptoms of interest were conducted. Previously reported associations of delusions with increased rates of extra-list intrusions and false recognitions of words might have been influenced by the overlap that delusions present with verbal hallucinations and thought disorganization. Our data, in contrast, revealed that, in the female patient sample, delusions were associated with decreased rates of false recognitions and, at a trend level, with decreased rates of intrusions. Word frequency did not have any impact on the inverse association with response bias. Semantic organization of the list, however, appears to be a relevant factor, as expected, since the inverse association with response bias was only observed for the two non-organisable lists. The meaning of a more conservative response bias in women as a function of delusion ratings is unclear. In any case, the pattern of associations of false recognitions with delusions, entirely distinct from that observed with verbal hallucinations, suggests that delusions are not related to impaired self-monitoring of inner speech. Although delusions have been found to be associated with external misattributions of self-generated speech (Allen et al., 2006; Johns et al., 2006; Anselmetti et al., 2007; Costafreda et al., 2008), the confusion might stem from reasoning abnormalities rather than from self-monitoring failure. Alternatively, only certain types of delusions –those resulting from passivity experiences– might be linked to self-monitoring failure (Keefe et al., 2002). The other types of delusions are more likely to be underpinned by distinct mechanisms such as theory-of-mind deficit, image of the self as vulnerable, and reasoning bias (Salvatore et al., 2012; Jolley et al., 2014).

### Thought Disorganization

Associations with thought disorganization were observed in the male patient subsample. Similar to verbal hallucinations, thought disorganization was associated with increased rates of extra-list intrusions in free recall, in agreement with previous studies (Brébion et al., 1999; Subotnik et al., 2006; Fridberg et al., 2010), and increased rates of false recognitions of non-target words, corroborating Moritz et al. (2003, 2005) and Ragland et al. (2003). Our data suggest that these associations are authentic since they remain significant when verbal hallucination score was entered in the regression analysis. Thus, both thought disorganization and verbal hallucinations appear to be associated with increased rates of commission errors in verbal memory. However, the examination of each type of list in the recognition task reveals that the association of these two clinical symptoms with the response bias did not follow identical patterns, suggesting distinct mechanisms. Thought disorganization has been related to lack of inhibition and aberrant spreading of semantic information (Kreher et al., 2008; Soriano et al., 2008; Kiefer et al., 2009). One might postulate that patients with thought disorganization are particularly prone to making semantic associations when reading the lists, and that their extra-list intrusions and false recognitions consisted mostly of semantic associates of the target words rather than of unrelated misattributed inner speech.

### Negative Symptoms

As expected, negative symptoms were associated with decreased rates of false recollection, although again this was observed only in male patients. An inverse association between extra-list intrusions and negative symptoms had already been reported in a few studies (Brébion et al., 1999, 2009; Turetsky et al., 2002; Heinrichs and McDermid Vaz, 2004). The inverse association with response bias corroborates our previous findings (Brébion et al., 2005) and those of Paz-Alonso et al. (2013), although it was only observed in the high-frequency list and at a trend level of significance. We had previously demonstrated that negative symptoms were also associated with a more conservative response bias in recognition tasks involving either pictures (Brébion et al., 2007) or a mixture of words and pictures (Brébion et al., 2002, 2008). These associations with decreased rates of commission errors might be explained by enhanced inhibition processes in patients with negative symptoms.

### Continuum

#### Hallucination Proneness

A few studies have shown that various types of verbal memory error reflecting impaired monitoring of inner speech were also associated with non-clinical hallucinations (Rankin and O’Carroll, 1995; Larøi et al., 2004; Brébion et al., 2010; Sugimori et al., 2011; Kanemoto et al., 2013). Our findings confirm these observations. In our healthy participant sample the number of extra-list intrusions was linked to hallucination proneness, which replicates our previous study (Brébion et al., 2010). The association, however, did not pertain exclusively to men as was the case in patients, but rather to a subsample of participants who experience intrusions. With respect to the increased rate of false recognitions, its association with hallucination proneness corroborates previous studies of non-clinical hallucinations (Brébion et al., 2010; Sugimori et al., 2011; Kanemoto et al., 2013). Further, the pattern of associations in the healthy sample was identical to that demonstrated by the patient group: the association of response bias with hallucination proneness emerged only in men, and it was significant for both the high-frequency and the semantically organisable lists, but was not observed for the low-frequency list. These converging findings and the comparable patterns of associations with response bias in our two participant groups suggest that misattribution of inner speech to an external source similarly underlies clinical and non-clinical verbal hallucinations, which supports the notion of a continuum from normality to pathology as far as this mechanism is concerned. Our data are compatible with a review of the cognitive mechanisms of auditory/verbal hallucinations, which revealed that intrusive memories and thoughts, as well as poor inhibitory control, contribute to both clinical and non-clinical verbal hallucinations (Badcock and Hugdahl, 2012). They are also in agreement with a meta-analysis (Brookwell et al., 2013) indicating that the externalization of internally generated events contributes in similar manner to both clinical and non-clinical hallucinations.

#### Negative Schizotypy

Social anhedonia has been shown to be a predictor of transition to psychosis in high-risk populations (Velthorst and Meijer, 2012; Wang et al., 2014). In our healthy sample an inverse association between social anhedonia and number of extra-list intrusions was observed, but it did not reach statistical significance. However, a significant association between social anhedonia and decreased rates of false recognitions emerged in healthy men, corroborating the inverse association with negative symptoms in the male patients. As far as we know, the only comparable studies of healthy participants are those of Sugimori et al. (2011) and Kanemoto et al. (2013) who did not uncover any inverse association between negative schizotypy and false recognitions. Social anhedonia might be more specifically involved in this relationship than is negative schizotypy. Our data suggest that the opposite association of positive and negative symptoms with memory errors, reported in this patient sample and a few others (Stirling et al., 2001; Brébion et al., 2002, 2012; Chiu et al., 2016), may also be obtained in healthy participants. Further, this might not be restricted to memory errors since atypicality of produced exemplars was found to be increased along thought disorganization and decreased along certain negative symptoms in both a schizophrenia (Brébion et al., 2013a) and a schizotypy (Minor et al., 2011) sample. Decreased rates of commission errors and reduced atypicality may both be interpreted as stemming from strengthened inhibition of semantic spreading. Future work should investigate whether an inverse association with certain aspects of negative schizotypy also exists for memory errors that stem merely from self-monitoring failure, such as confusion between said and thought information, and misattribution of one’s own verbal production to the experimenter.

## Limitations and Conclusion

A limitation of our work is that the patient and healthy samples were not matched with respect to education level and verbal IQ, and these demographic variables might influence the relationships with hallucinations. Antipsychotic medications might also have influenced the associations in patients. Further, in both the healthy and the patient groups, the female subsample was less sizeable than was the male subsample. The unexpected inverse associations of intrusions and response bias with delusions in the female patients might be due to the influence of unrepresentative data. Meanwhile, the absence of the expected positive associations with hallucinations in female participants from both groups might stem from lack of statistical power. Therefore, no strong conclusions regarding gender differences in the observed associations with hallucinations can be drawn. Nonetheless, our findings support the alleged role of misattributed inner speech in the formation of verbal hallucinations, and argue in favor of a continuum from normality to pathology in the disruption of self-monitoring of inner speech.

## Authors Contributions

GB designed the study, analyzed the data, and wrote up the results. CS-O, SO, and JU contributed to writing up the protocol and discussing the results. MR and LN contributed to the cognitive and clinical assessment of the participants. All authors contributed to and have approved the final manuscript.

## Conflict of Interest Statement

The authors declare that the research was conducted in the absence of any commercial or financial relationships that could be construed as a potential conflict of interest.
